# Development and evaluation of culture media based on extracts of the cyanobacterium *Arthrospira platensis*

**DOI:** 10.3389/fmicb.2022.972200

**Published:** 2022-08-09

**Authors:** Elaheh Kheirabadi, Javier Macia

**Affiliations:** ^1^Department of Medicine and Life Sciences Universitat Pompeu Fabra, Barcelona, Spain; ^2^BioInspired Materials Company, Barcelona, Spain

**Keywords:** cyanobacterium extracts, microbiology, *Arthrospira platensis*, culture media, animal and vegetal component free media, bacterial cellulose, not animal-derived culture medium, industrial biomolecules

## Abstract

Continuous advances in the fields of industrial biotechnology and pharmacy require the development of new formulations of culture media based on new nutrient sources. These new sources must be sustainable, high yielding, and non-animal-based, with minimal environmental impact. Thus, culture media prepared from cyanobacterial extracts can be an interesting alternative to the current formulations. In this study, we prepared various minimal formulations of culture media using the extracts of *Arthrospira platensis*, and analyzed the efficiency of these formulations, based on their effect on the production of biomass and molecules of industrial interest, using different types of bacteria. All media formulations prepared in this study showed better performance than conventional media, including those based on animal ingredients. Thus, based on their versatility and high-yielding capacity, we conclude that culture media prepared from cyanobacterial extracts are a good alternative to conventional media for meeting the current demands of the cosmetic and pharmaceutical industries.

## Introduction

Technological advancements are continuously needed for a wide range of applications in biotechnological and pharmaceutical industries, such as the production of vaccines (Plotkin et al., 2017), blood and blood components (Haldar et al., 2019), cell lines for cell therapies (Thakar et al., 2020), gene therapies (Gonçalves and de Paiva, 2017; Papanikolaou and Bosio, 2021), and recombinant therapeutic proteins (Dingermann, 2008; Qarawi et al., 2019). These advancements require new, high-yielding, and cost-effective formulations of culture media based on sources that are sustainable, have a zero or negative carbon footprint, and are preferably vegan, especially if used in the cosmetic industry (Pathak and Martirosyan, 2012; Pathak and Singhal, 2013).

Soy is currently the most widely used nutrient in the production of animal compound-free media. Although soy shows excellent performance, the exponential increase in soybean production worldwide is generating a high negative environmental impact (da Silva et al., 2021). Therefore, many efforts have been made in recent years to identify new sources of nutrients, especially those lacking components of animal origin, which could be used to prepare new culture media (Arulanantham et al., 2012; Batista and Fernandes, 2015; Narh et al., 2018).

An alternative to animal component-based media, with great potential, are the extracts of cyanobacteria, such as *Arthrospira* spp. Cyanobacteria have long been known for their rich nutritional profile (Schmidt and Jónasdóttir, 1997; Bahlol, 2014), and their commercial use as a food supplement for human and animal health is becoming more widespread. Not only can these photosynthetic microorganisms be cultivated on a large scale in a small area at low cost, but they also absorb atmospheric CO_2_ during growth, thus having a positive environmental impact. Despite these advantages, there are currently no commercially available culture media prepared from the hydrolyzed extracts of cyanobacteria. Moreover, little research has been done to explore the potential of cyanobacteria as a raw material for the development of new culture media (Jeong et al., 2021).

In this study, we explored the performance of cyanobacterial extract (CE)-based formulations in terms of their (i) versatility for the growth of diverse organisms; (ii) biomass production yield; (iii) protein expression; and (iv) production of molecules of industrial interest, with a primary focus on the expression of recombinant proteins in *Escherichia coli* and the production of bacterial cellulose, one of the most interesting biomaterials owing to its unique properties (Betlej et al., 2021) that can be applied in multiple industrial and biomedical applications (Chang and Chen, 2016; Picheth et al., 2017). To this end, the set of strains analyzed include (i) bacteria from very different environments and living in different atmospheric conditions, i.e., aerobic and anaerobic strains, (ii) pathogenic and non-pathogenic strains, and (iii) strains of industrial interest in various fields, from protein production to treatment of plant diseases in agriculture.

The minimal media analyzed in this study were composed of either the CE alone or a combination of CE and yeast extract (YE).

## Materials and methods

### Media composition

All culture media analyzed in the study contained, as the main ingredient, the hydrolyzed extract of *Arthrospira platensis* (Ficogenic®), and were supplied as a concentrated liquid by BioInspired Materials Company (BIOM, Barcelona, Spain). Composition of *A. platensis* extract was analyzed in collaboration with the Eurofins Company. Results are shown in Supplementary Table S1. The composition analysis showed significant amounts of amino acids, sugars, and carbohydrates indicating, *a priori*, a composition rich enough to cover the nutritional needs of diverse types of microorganisms.

Different formulations (Table 1) were prepared by directly diluting the CE in distilled water with or without 0.5% (w/v) YE. Then, the formulations were sterilized followed by autoclaving at 121°C for 20 min.

**Table 1:** Composition of minimal media prepared in this study.

Medium	Composition^1^
P1	30% CE
P2	20% CE
P3	10% CE
P4	5% CE
P5	2.5% CE
M1	30% CE + 0.5% YE
M2	20% CE + 0.5% YE
M3	10% CE + 0.5% YE
M4	5% CE + 0.5% YE
M5	2.5% CE + 0.5% YE

1 CE, cyanobacterial extract (v/v); YE, yeast extract (w/v).

The performance of culture media was evaluated by comparing the growth of different microorganisms in a test culture medium and on a reference culture medium, which was selected based on the specifications of the American Type Culture Collection (ATCC). The different microorganisms, reference media, and culture temperatures analyzed in this study are listed in Table 2. It should be noted that these bacteria originate from very different environments and have different nutritional requirements.

**Table 2:** List of microorganisms and corresponding reference culture media (according to ATCC) analyzed in this study.

Bacterial species	Bacterial strain	Standard medium	Culture conditions		Reference medium
			Atmosphere	Temperature (°C)	
*Escherichia coli*	DH5α	LB	Aerobic	37	Sigma-Aldrich L3522
*Bacillus subtilis*	PY79	Nutrient broth		37	Sigma-Aldrich 70,122
*Bacillus thuringiensis*	Kurstaki PB-54	Nutrient broth		30	Sigma-Aldrich 70,122
*Vibrio harveyi*	ATCC 14126	Photobacterium broth (PbB)		26	Sigma-Aldrich 38,719
*Listonella anguillarum*	ATCC 43306	Marine broth (MB)		18	Sigma-Aldrich 76,448
*Cupriavidus necator*	ATCC 17697	Nutrient broth		26	Sigma-Aldrich 70,122
*Bacteroides fragilis*	ATCC 25285	Trypticase soy broth with 5% defibrinated sheep blood (TSB + B)	Anaerobic	37	Lab prepared
*Cutibacterium acnes*	KPA171202	Brain heart infusion broth (BHI)		37	Sigma-Aldrich 53,286

Some of the reference media were prepared in the laboratory (Supplementary material) using commercially available ingredients including yeast extract (Sigma-Aldrich Y1625), glucose (Sigma-Aldrich G8270), tryptone (Sigma-Aldrich T9410), beef extract (Sigma-Aldrich B4888), sodium acetate (Sigma-Aldrich S2889), CaCO_3_ (Sigma-Aldrich 239216), peptone (VWR J636), mannitol (Labkem MANL-00A-500), Tryptic Soy Broth (Sigma-Aldrich 22092), and defibrinated sheep blood (Thermo Scientific SR0051C).

Other media used in this study were purchased from commercial manufacturers: SOC Medium (Thermo Fisher 15544034) and 2YT (Sigma-Aldrich Y2377).

### Bacterial cultures

All cultures were prepared by inoculating a single colony into the corresponding medium, and maintained at the proper temperature (Table 2) with orbital shaking at 200 rpm for 20 h.

For the preparation of cultures from lyophilized *E. coli*, 1 mg of lyophilized powder was resuspended in 50 μl of PBS. Next, 180 μl of each culture medium was loaded into the wells of a 96-well plate. Finally, each well was inoculated with 2 μl of the *E. coli* cell suspension in PBS. Time course measurements were done with Synergy HXT-Multi-Mode Reader (BioTek Instruments, United States) at 37°C with continuous orbital shaking at 200 rpm for 20 h.

The AnaeroGen™ 2.5 l system (Thermo Scientific) was used to grow microorganisms in an anaerobic atmosphere.

### Recombinant protein expression

To evaluate the expression of recombinant proteins, *E. coli* DH5α strain was transformed with the plasmid pSB1C3, which contained the *Green Fluorescent Protein* (*GFP*) gene under the control of a constitutive promoter (see Supplementary Table S2 for details).

### Optical density and fluorescence measurements

To measure the optical density (OD) and fluorescence of different cultures, a 4 ml volume of each culture medium was prepared. After 20 h of culture at the appropriate temperature with shaking, 1 ml of each culture was centrifuged for 2 min at 13,000×*g*. The supernatant was subsequently removed, and the pellet was resuspended in phosphate-buffered saline (PBS). Finally, 200 μl of each culture was loaded onto a 96-well plate. The OD of each culture was measured at a wavelength of 600 nm. Fluorescence measurements were recorded at emission and excitation wavelengths of 509 ± 9 nm and 475 ± 9 nm, respectively, with Synergy HXT-Multi-Mode Reader (BioTek Instruments, United States). Experimental data were collected with the Gen5 software package and analyzed with Microsoft Excel 2019. The fluorescence of each culture was calculated using the following equation:


(1) $$GFP=\frac{GFP-GFP_{0}}{OD-OD_{0}}$$

where GFP_0_ and OD_0_ represent the fluorescence and OD values of PBS (blank), respectively.

### DNA transformation protocol

For DNA transformation, 120 μl of a culture of competent *E. coli* DH5α was mixed with 0.2 μl of DNA and maintained on ice for 20 min. To induce heat shock, each cell culture was exposed to a high temperature (45°C) for 45 s. Subsequently, the cells were incubated on ice for 10 min. Then, 20 μl of the transformed cells were transferred to Eppendorf tubes containing 480 μl of the different media, and cultured for 1 h at 37°C with constant shaking at 350 rpm. Finally, the contents of each tube were spread on a Petri dish containing LB-agar medium and stored at 37°C overnight.

### *Escherichia coli* lyophilization

A culture of *E. coli* was prepared in 10 ml of LB medium overnight at 37°C. Subsequently, the culture was centrifuged and the supernatant was removed. The pellet was resuspended in 10 ml of 10% (w/v) sucrose solution. The suspension was frozen with liquid nitrogen and then placed in a lyophilizer (Telstar LyoAlfa 15) for 48 h.

### Statistical analysis

Results shown in figures are the mean of three independent experiments. Error bars are the standard deviation of the three independent experiments. The statistical significance of the results obtained using the different CE-based media analyzed vs. the results obtained using the reference medium was determined by the value of p in the Student’s *t*-test. The range of the value of *p* is shown in each figure according to ^*^p < 0.05, ^**^p < 0.01, ^***^p < 0.001, and – for no statistical significance.

## Results

### Efficiency of biomass production using CE-based media

One of the important aspects of many biotechnological applications at the industrial level is to achieve high densities of microorganisms in bioreactors. For example, the production of biopesticides (Ernandes et al., 2013; Morales-Borrell et al., 2020), biofertilizers (Mukhtar, 2018), and lactic ferments (Coelho et al., 2011), among other products, requires optimized media for producing large amounts of biomass. As a result, the industry continues to seek new sources of nutrients. To reach the objective of increasing biomass production, formulations with specific compositions for each type of microorganism are generally developed. These media formulations are based on the combination of different carbon and nitrogen sources of both animal and vegetable origin. The first objective of this study was to evaluate the efficiency of CE-based media as a general nutritional basis for biomass production, regardless of the particular characteristics of each type of microorganism. This represents a significant simplification in the formulation of the new media, based principally on a single source of nutrients, which, in turn, is associated with simplifying production processes and reducing production costs.

**Figure 1:**
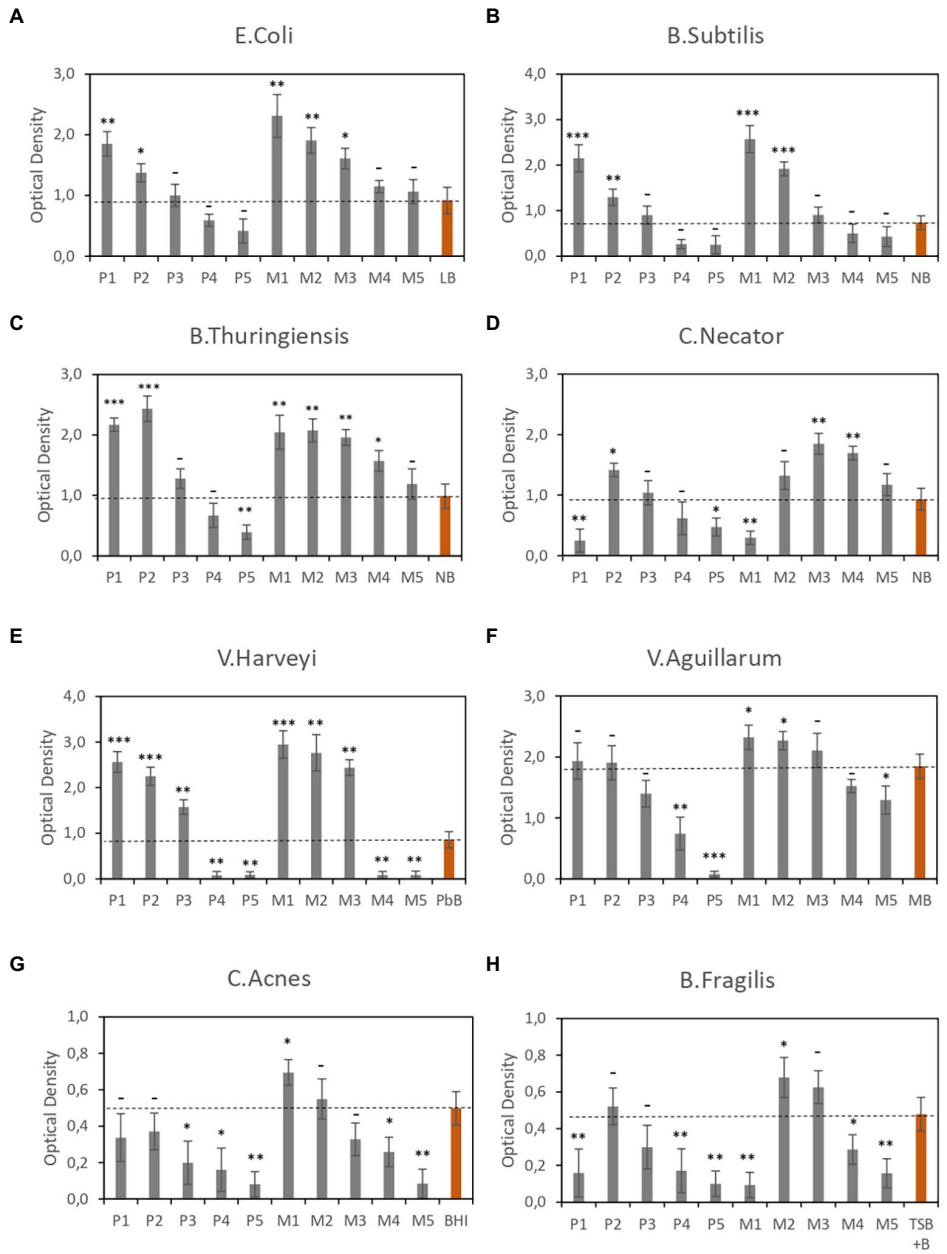
Optical density of bacterial cultures in different CE-based media. **(A–F)** Aerobic cultures. **(G,H)** Anaerobic cultures. Data are the average of three independent experiments. Red bars correspond to the reference media. Error bars are the standard deviation of three independent experiments. *p*-values ^*^p < 0.05, ^**^p < 0.01, ^***^p < 0.001, and ^–^*p* ≥ 0.05.

To evaluate the efficiency of CE-based media, we performed multiple growth assays using all formulations (Table 1) with different types of bacteria (Table 2). The culture conditions, in terms of oxygen level (aerobic or anaerobic) and temperature, varied with the type of microorganism. The growth of each microorganism was evaluated on the test formulation and the reference medium (Table 2) under the same conditions. The results of cell growth assays, measured in terms of OD, are shown in Figure 1. Figures 1A–F show the optical density measured in different types of aerobic bacterial cultures using the CE-based media described in Table 1, whereas Figures 1G,H show the optical density in two different types of anaerobic bacterial cultures, for each CE-based media.

In general, the experimental results show a direct relationship between CE concentration and biomass production in all strains analyzed, showing higher OD values on formulations containing >10% CE than on the reference media, indicating a higher rate of cell growth. It should be noted that media composed of both CE and YE performed better than media composed of CE alone. This trend was observed with all microorganisms studied, regardless of their intrinsic characteristics.

However, in some strains, this ratio is not satisfied at higher CE concentrations, i.e., 30%. A possible cause could be elevated osmotic stress associated with these media with elevated concentrations. This would be consistent with previous results. For example, high salinity has been reported to negatively impact strains like *Bacillus thuringiensis* (Jiang et al., 2006) or *C. Necator* (Passanha et al., 2014).

Despite this negative effect at higher CE concentrations in some strains, the CE-based media contained no animal-derived components; nonetheless, the levels of biomass production obtained using CE-based media were better than those obtained using reference media such as nutrient broth (for *Bacillus subtilis* and *B. thuringiensis*; which contains beef extract), brain heart infusion broth (for *Cutibacterium acnes*), and Trypticase soy broth with 5% defibrinated sheep blood (for *Bacteroides fragilis*). This is a remarkable result because some of these strains are characterized by being difficult to grow, indicating that CE-based media are a good alternative to animal fluid-based media.

### Evaluation of CE-based culture media as an alternative to enriched media

Given the positive effects of CE-based media on cell growth, we compared the performance of these media with that of two enriched media (2YT and SOC), which are commonly used for cell recovery post-transformation.

In both cases, the fragility of the cells can affect their survival rate. In these circumstances, the media composition plays a key role in the rate of cell growth. This is particularly relevant in the case of massive use of lyophilized microorganisms, for instance as biofertilizers in agriculture, where rehydration of lyophilized cells before their application improves functional properties (Arellano-Ayala et al., 2021).

In order to evaluate the efficiency of CE-based media in DNA transformation, *E. coli* strain was transformed with a plasmid constitutively expressing GFP. Specifically, the pSB1C3 plasmid containing the *GFP* gene under the control of the constitutive J23100 promoter was constructed and transformed into *E.coli* (see Supplementary material for details).

**Figure 2:**
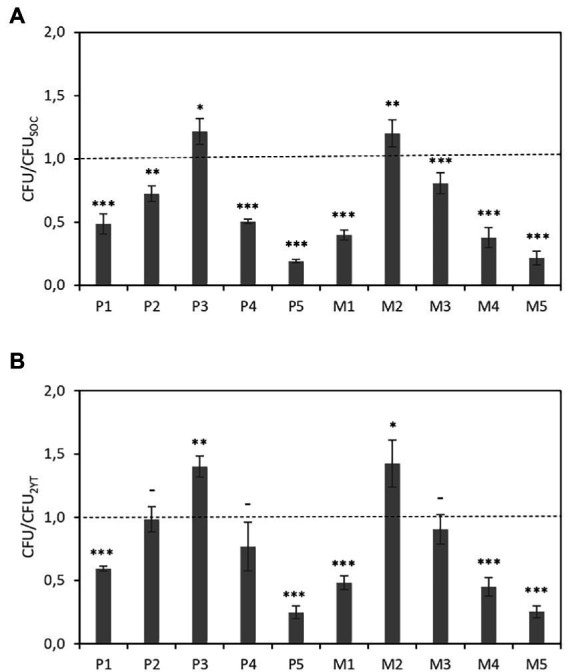
DNA transformation efficiency of bacterial grown in different CE-based media after heat shock. **(A)** The CFUs of cultures in CE-media are expressed relative to those in SOC medium. **(B)** The CFUs of cultures in CE-media are expressed relative to those in 2YT medium. Data are the average of three independent experiments. Error bars are the standard deviation of three independent experiments. *p*-values ^*^p < 0.05, ^**^p < 0.01, ^***^p < 0.001, and ^–^*p* ≥ 0.05.

The number of colony-forming units (CFUs) was counted to determine the effect of the culture medium on the transformation efficiency. Figure 2A shows the CFUs of cultures in CE-media (CFU_CE medium_) relative to those in SOC medium (CFU_SOC_), i.e., CFU_CE medium_/CFU_SOC_. Similarly, Figure 2B shows the CFUs relative to those in 2YT medium, i.e., CFU_CE medium_/CFU_2YT_. As figures show, the transformation efficiencies obtained using P3 and M2 media were 20% higher than those obtained using SOC and 40% higher than those obtained using 2YT.

**Figure 3:**
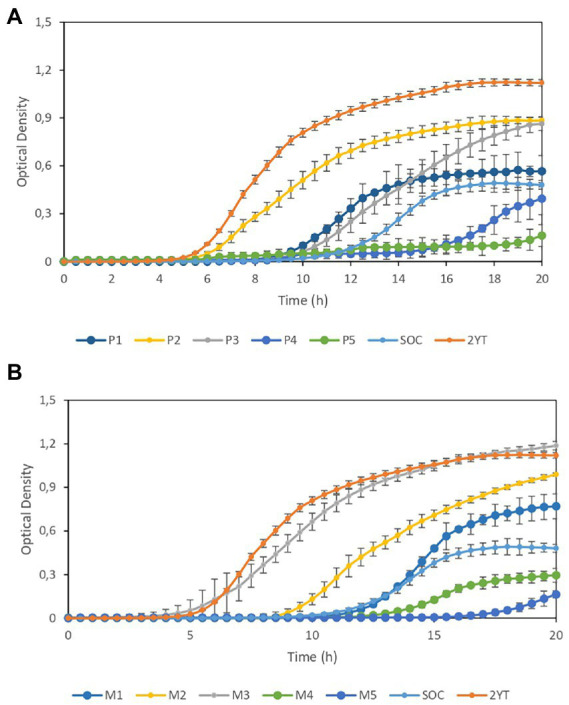
Growth curves of lyophilized *Escherichia coli*-derived cultures. **(A)** Comparison of the growth dynamics of cultures in P1–P5 media and those in SOC and 2YT media. **(B)** Comparison of the growth dynamics of cultures in M1–M5 media and those in SOC and 2YT media. Error bars are the standard deviation of three independent experiments.

Finally, we analyzed the growth of lyophilized *E. coli* in CE-based media that produced a higher yield in cell growth assays along with 2YT and SOC as reference media. Figure 3A shows the growth curves of lyophilized *E. coli*-derived cultures in P1–P5 media compared with the growth dynamics of cultures in SOC and 2YT. Similarly, Figure 3B compares the growth dynamics of cultures in M1-M5 with those in SOC and 2YT. Although the 2YT medium showed the best results, the performance of M3 medium was similar to that of 2YT, indicating that M2 is a good alternative to 2YT. On the other hand, media containing >10% CE exhibit better performance than SOC cultures.

Experimental results show that combining CE with YE is significantly more effective than CE-only formulations. However, it is worth mentioning that increasing concentrations of CE does not imply better performance neither in terms of transformation efficiency nor in the growth of lyophilized *E. coli*-derived cultures, similar to the previous observed effect on growth of some of the strains analyzed.

### Efficiency of the production of molecules of interest using CE-based culture media

One of the areas where the search for new sources of nutrients is more intense is the industry dedicated to producing biomolecules and biomaterials. Here, the formulation of new sustainable, low-cost, and high-yielding culture media is challenging.

To evaluate the potential of CE-based media as a substrate for the industrial-scale production of biomolecules, two case studies were considered: the production of recombinant proteins in *E. coli* and production of bacterial cellulose using *Gluconacetobacter xylinus* (strain ATCC 23767) and *Komagataeibacter sucrofermentans* (strain ATCC 700178).

Constitutively expressed GFP was used as the recombinant protein (see Supplementary material). A preculture of *E. coli* was prepared in LB medium and maintained at 37°C overnight on a shaker at 300 rpm. Subsequently, 4 ml of each culture medium (Table 1) and LB (reference medium) was prepared. Finally, 2 μl of the preculture was used to inoculate these media, and the inoculated media were incubated for 20 h at 37°C and 300 rpm.

**Figure 4:**
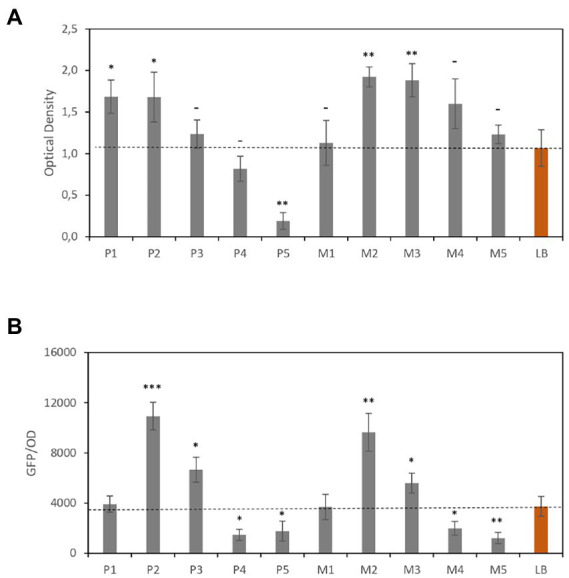
Recombinant GFP expression levels in *Escherichia coli*. **(A)** Optical densities of *E. coli* cultures. **(B)** GFP fluorescence normalized to optical density. Red bars correspond to the reference media. Data are the average of three independent experiments. Error bars are the standard deviation of three independent experiments. *p*-values ^*^p < 0.05, ^**^p < 0.01, ^***^p < 0.001, and ^–^*p* ≥ 0.05.

Figure 4A shows the OD and Figure 4B shows GFP/OD data. Compared with LB, the P2, P3, M2, and M3 media showed higher protein yield. As previously observed, the media with the highest CE concentrations (P1 and M1) showed lower efficiency, which was comparable with that obtained using LB. However, a reduction in CE concentration led to a significant increase in both cell number (OD value) and GFP signal per cell.

The second case study focused on the production of bacterial cellulose, which is a polymer of great interest. Intensive research is currently being conducted to develop new media that could increase the production of bacterial cellulose (Gorgieva and Trček, 2019; de Fernandes et al., 2020; Zhong, 2020; Wahid et al., 2021). In this study, precultures of both *G. xylinus* and *K. sucrofermentans* were prepared overnight at 27°C using mannitol broth and YGC medium, respectively (see Supplementary material for media composition). Subsequently, Petri dishes containing 20 ml of media (Table 1), in addition to control plates containing mannitol broth or YGC medium, were prepared and inoculated with 2 μl of the corresponding preculture. It should be noted that CE-based media were supplemented with 5% (w/v) glucose. The plates were incubated at 27°C for 14 days in static culture.

**Figure 5:**
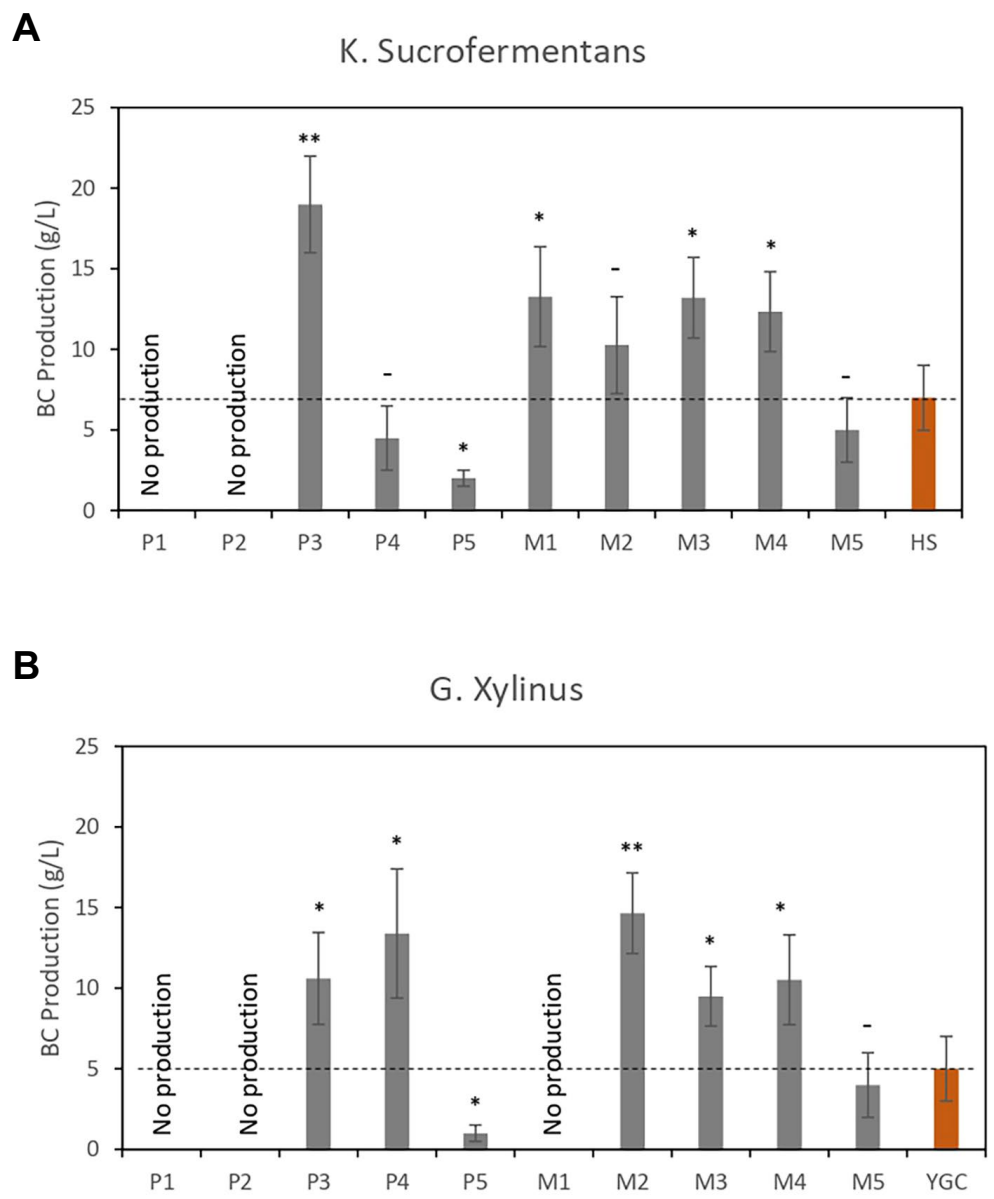
Dry weights of bacterial cellulose (BC; g/L) from cultures grown in different CE-based media. **(A)** BC produced by *K. sucrofermentans*. **(B)** BC produced by *G. xylinus*. Red bars correspond to the reference media. Data are the average of three independent experiments. Error bars are the standard deviation of three independent experiments. *p*-values ^*^p < 0.05, ^**^p < 0.01, and ^–^*p* ≥ 0.05.

The bacterial cellulose membranes produced were collected, immersed in 0.1 M NaOH solution, and heated to 90°C with string for 2 h to remove cellular debris (Hashim et al., 2021). Then, the membranes were washed several times with distilled water to remove excess NaOH until the pH decreased to 6.5. The purified membranes were lyophilized for 2 days and their dry weight was determined. Figure 5A shows the production of BC (in g/L) in *K. sucrofermentans* cultures, whereas Figure 5B shows the results obtained in *G. Xylinus* cultures. The results showed that bacterial cellulose membranes were not formed in culture media containing high CE concentrations and lacking YE. The mechanism that inhibits the formation of bacterial cellulose remains unclear but could be related to media osmolality. However, a significantly higher amount of bacterial cellulose was produced with both strains in media containing low CE concentrations compared with reference media, making the CE-based media especially valuable for bacterial cellulose production.

## Discussion

Many efforts are currently underway to find new sources of nutrition for microbiology applications, with a particular focus on finding alternatives that do not use animal-derived components. The industry currently demands new media formulations that offer high yields at low cost, can be produced in a sustainable manner, and thus have a minimal environmental impact. Compared with other nutritional sources, cyanobacteria contain the highest levels of proteins, amino acids, minerals, pigments, vitamins, and polysaccharides (Panjiar et al., 2017). It is worth mentioning that cyanobacteria can be produced in an ecological and sustainable manner and at low cost just using industrial waste products and atmospheric CO_2_ (Zhu et al., 2020; Lim et al., 2021). Nonetheless, little research has been conducted in the field of microbiology on the use of cyanobacteria as a nutritional source for the production of culture media.

This study analyzed the efficiency of new formulations of culture media based on the extract of *Arthrospira platensis* as the main ingredient.

First, these media were evaluated for biomass production using both aerobic and anaerobic bacteria, and were compared with different reference media containing ingredients of animal origin, such as beef extract, brain heart infusion, or blood. Increasing biomass production is one of the most important challenges for multiple industries, such as probiotic or lactic ferments producers among others.

All CE-based media analyzed showed better results, in terms of cell growth, than the corresponding reference media. Importantly, best results were also obtained with microorganisms requiring ingredients of animal origin in the standard media. These results indicate that CE-based media are a good alternative to animal component-based media because of their high performances and cost-effective production and have higher consumer acceptance, especially in cosmetic and pharmaceutical industries, where new animal ingredient-free and ecologically produced media are preferred.

Related with the positive effects of CE-based media in cell growth, two interesting applications have been explored, namely the effects of CE-based in the DNA transformation efficiency and the in the culturing of lyophilized cells.

Increasing DNA transformation efficiency is appealing for multiple industrial applications. For instance, recombinant DNA technology plays a vital role in improving health conditions by developing new vaccines and pharmaceuticals or in the production of diverse industrial products (Adrio and Demain, 2010).

Regarding the use of lyophilized microorganisms, it should be noted that lyophilization is the optimal method for preserving microorganisms and their use is of great interest in sectors such as agriculture, where the use of lyophilized microorganisms for the treatment of plant pathologies, to promote plant growth and enhance biotic and abiotic stress resistance is increasingly widespread (Ma et al., 2021). It has been reported that rehydration of lyophilized cells before their application improves functional properties (Arellano-Ayala et al., 2021).

For both cases, DNA transformation and rehydration of lyophilized cells, enriched media, such as SOC or 2YT, are required. This study demonstrates that CE-based media can be used for both purposes, improving the efficiency of DNA transformation and achieving similar or better performance in the culturing of lyophilized cells when compared with SOC and 2YT. Hence, CE-based media could represent an optimal alternative to the standard media, for sustainable, ecological, and vegan production processes.

Finally, the CE-based media showed great potential for the production of biomolecules of industrial interest, especially recombinant proteins in *E. coli* and bacterial cellulose production in *G. xylinus* and *K. sucrofermentans*. Increasing the production of proteins and biomaterials such as BC is a challenge for industry. Many efforts have been devoted to design and optimize culture media combining high performance with cost-effectiveness. In both cases, CE-based media have shown better performance compared to standard media. The uses of CE-based media may represent a significant advance toward an improved industrial production of recombinant proteins and biomaterials.

However, it is necessary to dedicate more efforts to the design and optimization of new formulations of CE-based media. It has been observed that the increase in the concentration of CE in the formulations of the culture media is not always directly associated with an increase in their performance. Actually, in some cases, increasing CE concentrations beyond a critical level has a negative effect. Although the reason for this behavior is not clear, these results suggest that the osmotic stress produced by high concentrations of CE can affect the media efficiency, pointing out that it is necessary to optimize the formulation of the medium for each type of bacteria.

In summary, this study shows that CE-based media can serve as efficient sources of nutrition for a wide range of bacteria originating from different environments, and can be used in various types of applications such as the production of biomass and molecules of interest. These results, together with the low cost of production of cyanobacteria using industrial waste products and atmospheric CO_2_, make cyanobacteria a very attractive alternative for the formulation of new animal compound-free media.

Despite the great potential of CE-based media, their applications have not been explored thoroughly. Future work should focus on exploring the potential use of CE-based media in other cellular strains, such as mammalian cell growth.

## Data availability statement

The raw data supporting the conclusions of this article will be made available by the authors, without undue reservation.

## Author contributions

JM conceived the experiments and wrote the manuscript. EK and JM performed the experiments. All authors contributed to the article and approved the submitted version.

## Funding

Funding for this study and for the open access charge was in the form of grants from the Industrial Doctorate Program funded by the Generalitat de Catalunya. This work was supported by “Unidad de Excelencia María de Maeztu,” funded by the MCIN and the AEI (DOI: 10.13039/501100011033). Ref: CEX2018-000792-M. Ficogenic® development, production and characterization was supported by ACCIO, Generalitat de Catalunya.

## Conflict of interest

The cyanobacteria extracts used in this study (Ficogenic®) were commercial products developed by the Biom Company, a spin-off of Univesitat Pompeu Fabra. This study was developed as part of an Industrial Doctorate Program under an agreement between the Universitat Pompeu Fabra and the Biom Company.

The authors declare that the research was conducted in the absence of any commercial or financial relationships that could be construed as a potential conflict of interest.

## Publisher’s note

All claims expressed in this article are solely those of the authors and do not necessarily represent those of their affiliated organizations, or those of the publisher, the editors and the reviewers. Any product that may be evaluated in this article, or claim that may be made by its manufacturer, is not guaranteed or endorsed by the publisher.
