# Supplementary material for: Development and evaluation of culture media based on extracts of the cyanobacterium Arthrospira platensis
Source: Front Microbiol. 2022 Aug 9;13:972200. doi: 10.3389/fmicb.2022.972200 (PMC9404535; doi:10.3389/fmicb.2022.972200)
Supplement: Supplementary file 1 [file Data_Sheet_1.PDF]

SUPPLEMENTARY MATERIALS

Development and evaluation of culture media based on extracts of the cyanobacterium *Arthrospira platensis*

Kheirabadi, Elaheh.[1,2], Macia, Javier.[1,*]

[1]Department of Medicine and Life Sciences, Universitat Pompeu Fabra, Barcelona Biomedical Research Park. Avda. Dr. Aiguader 88, 08003 Barcelona, Spain.

[2]BioInspired Materials Company, c\Valencia 359, 08009 Barcelona, Spain.

CE COMPOSITION

**Sugars**		**Amino acids**	
Fructose	<0.10 g/100 g	Alanine	0.423 g/100 g
Galactose	0.12 g/100 g	Arginine	0.373 g/100 g
Glucose	0.47 g/100 g	Aspartic acid	0.570 g/100 g
Lactose	0.14 g/100 g	Cystein +Cystine	0.0430 g/100 g
Maltose	<0.10 g/100 g	Glutamic acid	0.769 g/100 g
Sucrose	<0.10 g/100 g	Glycine	0.280 g/100 g
		Histidine	0.0823 g/100 g
**Carbohydrates**	4.6 g/100g	Hydroxyproline	<0.2 g/100 g
		Isoleucine	0.258 g/100 g
**Metals**		Leucine	0.425 g/100 g
Calcium (Ca)	2.3 mg/100 g	Lysine	0.279 g/100 g
Manganese (Mn)	<0.050 mg/100 g	Methionine	0.0870 g/100 g
Magnesium (Mg)	22 mg/100 g	Ornithine	<0.05 g/100 g
Zinc (Zn)	<0.050 mg/100 g	Phenylalanine	0.217 g/100 g
Potassium (K)	132 mg/100 g	Proline	0.238 g/100 g
		Serine	0.269 g/100 g
		Threonine	0.281 g/100 g
		Thyptophane	<0.1 g/100g

	Tyrosine	0.219 g/100 g
	Valine	0.312 g/100 g

Table S1. Compositional analysis of CE.

LAB PREPARED MEDIA

Tryptic Soy Medium with 5% Defibrinated Sheep Blood

Tryptic Soy Broth (BD 211825) …………….. 30.0 g

Sheep Blood (defibrinated)…………………... 50.0 mL

DI Water…………………………………….... 950.0 mL

Autoclave medium at 121ºC. Cool to ~47ºC.

Aseptically add 50 mL of room temperature defibrinated sheep blood.

YGC medium for bacterial cellulose production

Glucose....................50.0 g

Yeast extract...............5.0 g

$CaCO_3$ ....12.5 g

Agar.......................15.0 g

Distilled water.............1.0 L

Autoclave at 121C for 15 minutes.

Mannitol Agar/Broth

Yeast Extract……………………………………. 5.0 g

Peptone…………...…………………………….. 3.0 g

Mannitol……………...………………………….. 25.0 g

Agar (if required)……………......................... 15.0 g

DI Water………………………………………… 1000.0 mL

Autoclave medium at 121ºC.

GENETIC CONSTRUCT FOR GFP EXPRESSION

The plasmid pSB1C3 contains the gene encoding Green Fluorescent Protein (GFP) located downstream of a constitutive promoter. The genetic construct was created from genetic parts from the Registry of Standard Biological Parts using the Biobrick assembly method (http://parts.igem.org/). Specifically, the genetic construct was composed of the following genetic parts:

T14 + J23100 + RBS34 + GFP

where:

CODE	Description	Parts Registry ID
T14	Double terminator sequence	B0014
J23100	Strong constitutive promoter from Anderson's collection	J23100
RBS34	Strong Ribosomal Binding Site sequence	B0034
GFP	Green Fluorescence Protein	E0040

Table S2. Genetic components for GFP expression
